# Prevalence of Workplace Physical Violence against Health Care Professionals by Patients and Visitors: A Systematic Review and Meta-Analysis

**DOI:** 10.3390/ijerph17010299

**Published:** 2020-01-01

**Authors:** Yi-Lu Li, Rui-Qi Li, Dan Qiu, Shui-Yuan Xiao

**Affiliations:** Department of Social Medicine and Health Management, Xiangya School of Public Health, Central South University, Changsha 410078, Hunan, China; 176911065@csu.edu.cn (Y.-L.L.); 176911062@csu.edu.cn (R.-Q.L.); 166911058@csu.edu.cn (D.Q.)

**Keywords:** health care professionals, workplace violence, physical violence, meta-analysis

## Abstract

Workplace physical violence against health care professionals perpetrated by patients and visitors has been a persistent problem worldwide. Prevalence estimates varied vastly across studies and there was a lack of quantitative syntheses of prevalence studies. This review aimed to quantify pooled one-year prevalence estimates at the global and regional levels. A systematic literature search was performed in the databases of PubMed, PsycINFO, Web of Science, and Embase between 1 January 2000 and 8 October 2018. Studies providing information about one-year prevalence of self-reported workplace physical violence against health care professionals perpetrated by patients or visitors were included. Heterogeneity between studies was evaluated using Cochran’s chi-squared test (Cochran’s Q) and I^2^ values. Subgroup analysis and meta-regression were used to explore heterogeneity. A total of 65 eligible studies reported one-year prevalence estimates for 61,800 health care professionals from 30 countries. The pooled one-year prevalence of workplace physical violence against health care professionals perpetrated by patients or visitors was 19.33% (95% confidence interval (CI): 16.49–22.53%) and the overall heterogeneity was high across studies. We noted geographic and staff categories variations for prevalence estimates through subgroup analysis. The meta-regression showed that sample size, type of health care setting, and quality score were significant moderators for heterogeneity. One in five health care professionals experienced workplace physical violence perpetrated by patients or visitors worldwide annually. Practical intervention was needed to ensure safety of health care professionals.

## 1. Background

Workplace physical violence against health care professionals has been a persistent problem of health care environment worldwide [1,2]. Health care professionals include physicians, nurses, technicians, and other medical staff who are in direct contact with patients and visitors. In 2009, 10% of workplace assaults victims were health care professionals in the United States. [3]. The World Health Organization (WHO) defined workplace violence as the incidents where staff were abused, threatened, or assaulted in the circumstances related to their work [4]. Workplace violence had an explicit or implicit impact on employees’ safety, well-being, and health. Workplace violence can have multiple negative consequences that not only result in physical consequences [5], but also psychological consequences for health care professionals [6,7]. Additionally, workplace violence was associated with the intention to quit job [8], burnout [7], and decreased job satisfaction [9] among health care professionals. Those consequences of workplace violence can lead to decreased productivity and even affect the quality of care. Moreover, staff absence [10] and investment of defensive tactics (e.g., security guard and metal detector) [11] caused by workplace violence may virtually increase health costs. Therefore, workplace violence in health sectors affected not only the health care professionals themselves, but also the entire health care environment.

The WHO had classified workplace violence into physical violence and psychological violence. Physical violence was defined as physical force (e.g., beating, kicking, slapping, stabbing, shooting, pushing, biting, and pinching) against a person or groups that results in physical, sexual, or psychological harm [4]. Physical violence was the most serious type of violence against health care professionals in their workplace [12]. Health care professionals accounted for 1.2% of workplace homicide victims of the United States [3], and about 4.9–65% of health care professionals were physically injured in their workplace during an incident of workplace physical violence [5]. Work stress, patient expectations, and deteriorative patient–staff relationships were associated with workplace physical violence against health care professionals [13]. Before developing policies and interventions, it is important to understand the prevalence and severity of workplace physical violence against health care professionals.

In order to obtain relatively reliable pooled prevalence estimates, the research included in meta-analysis should be relatively consistent in definition. The definition of workplace physical violence was more consistent across studies [7,14,15]. Extensive studies have been conducted to explore the prevalence and severity of workplace physical violence against health care professionals perpetrated by patients and visitors. Estimates of one-year prevalence of workplace physical violence against health care professionals perpetrated by patients or visitors in general hospital ranged from 2.75% in Thailand [16] to 74.42% in the United States [17]. Only a few of the systematic reviews have synthesized the results of prevalence studies. Those systematic reviews mainly focused on high-risk health care sectors [18,19,20], specific professional group [21], or specific country [22]. There was still a need for a systematic review that included all health care sectors, diverse health care professional types, and multiple countries. In addition, workplace physical violence in health sectors was mainly perpetrated by patients and visitors [1,23]. However, co-workers or superiors may also be the perpetrators of workplace physical violence against health care professionals. The nature of workplace physical violence perpetrated by co-workers or superiors was distinctly different from that perpetrated by patients or visitors. However, numerous studies did not report who perpetrated the workplace violence. Most of the systematic reviews did not describe the identity of the perpetrators [18,19,20,21,22]. Therefore, the prevalence of workplace physical violence against health care professionals by patients and visitors is still not clear and there is a lack of quantitative synthesized results. Considering the limitations of previous research, our study aimed to synthesize the results of workplace physical violence against health care professionals by patients and visitors.

To address the need for global estimates of prevalence of workplace physical violence against health care professionals perpetrated by patients or visitors, we did a meta-analysis of relevant studies around the world. We also aimed to understand how the methodological characteristics (i.e., sample size, response rate, method of data collection, sampling method) and contextual factors (i.e., region, health care setting) influenced the variations in prevalence estimates. A systematic literature search was performed. Possible relevant studies were screened based on strict eligibility criteria. Quality of eligible studies was assessed. Quantitative synthesized one-year prevalence of workplace physical violence against health care professionals perpetrated by patients and visitors was obtained by the meta-analysis.

## 2. Methods

### 2.1. Search Strategy and Selection Criteria

This meta-analysis was performed according to the Preferred Reporting Items for Systematic Reviews and Meta-Analyses (PRISMA) guidelines (see Table S1, Supplementary Materials). The following four academic databases were searched between 1 January 2000 and 8 October 2018: PubMed, PsycINFO, Web of Science, and Embase. The search strategy was developed and adjusted for each database with a combination of free text and controlled vocabulary terms. The following search terms were used: “physical violence” (including “physical violence”, “workplace violence”, and “occupational violence”)”, “health care professional” (including “health care professional*”, “nurse*”, “doctor*”, “physician*”, and “health care worker*”), and “prevalence” (including “prevalence”, “incidence”, “cross-sectional”, and “cohort”). A full list of the search terms is provided in Table S2, Supplementary Materials. Additionally, reference lists of eligible studies were manually screened for any relevant studies. 

Studies were independently screened by two reviewers (Y.-L.L. and R.-Q.L.) using the eligibility criteria described below. Studies were included if they meet the following criteria: (i) provided one-year prevalence of self-reported workplace physical violence against health care professionals perpetrated by patients or visitors; or (ii) reported definition and measurement of workplace physical violence. We excluded studies if they met the following criteria: (i) included medical student, cleaning staff, clerk, security, or administrative staff as participants; (ii) did not report perpetrators of the workplace physical violence; (iii) reported response rate <20%, or no response rate was reported; or (iv) was conference abstract, report, review, meta-analysis, letters, pilot study, protocol, or qualitative study. Workplace physical violence against health care professionals included beating, kicking, slapping, stabbing, shooting, pushing, biting, and pinching against health care professionals in their workplace [4]. We included the studies only based on self-reported rather than record or monitoring data. To avoid bias in data synthesis, we included studies with the same prevalence period (one-year) for the meta-analysis. When findings from iterations of the same survey were reported, we included the publication that provided the most data.

### 2.2. Data Extraction and Quality Assessment

Two researchers (Y.-L.L. and R.-Q.L.) independently extracted relevant data from eligible studies and a third researcher (D.Q.) cross checked for accuracy. The following data were extracted: author, year of publication, country of study, sample size, categories of health care professionals, sampling method, method of data collection, response rate, type of health care setting, region of health care setting, and one-year prevalence estimates of workplace physical violence perpetrated by patients or visitors.

The methodological quality was assessed using the eight-item Loney criteria (see Table S3, Supplementary Materials). Studies satisfying one item will be given one point and an overall score was calculated. Therefore, the overall score ranged from zero to eight points, with higher scores indicating a higher degree of quality.

### 2.3. Data Analysis 

Statistical analyses were performed using the “meta” and “metafor” package of R version 3·5·2 (R Core Team, Vienna, Austria). Firstly, a normality test for the original study rates was performed to decide whether to transform the original rates. According to the normality testing results, a logit transformation method was used in this meta-analysis. Heterogeneity between studies was evaluated using Cochran’s chi-squared test (Cochran’s Q) and I^2^ values. The significant heterogeneity between studies was assumed when *p* < 0.1 or I^2^ > 50% [24]. A random effects model was adopted to calculate the pooled one-year prevalence of workplace physical violence against health care professionals perpetrated by patients or visitors if significant heterogeneity was observed across studies; otherwise, a fixed-effects model was adopted. To investigate the possible sources of heterogeneity and variations of prevalence estimates, subgroup analyses were conducted based on following categories: WHO regions (Western Pacific vs. European vs. eastern Mediterranean vs. Americas vs. African vs. South-East Asia); income classification of each country based on the World Bank classification (high-income vs. upper-middle-income vs. lower-middle-income vs. low-income countries); year of publication (2000~2010 vs. 2011~2018); sample size (≤500 vs. >500); response rate (≤50% vs. >50%); professional group (nurses vs. physicians); method of data collection (self-administered vs. face-to-face interview vs. telephone interview); gender (male vs. female); sampling method (all vs. random vs. convenience); type of health care setting (tertiary hospital vs. secondary hospital vs. primary care facilities vs. nursing home); region of health care setting (urban vs. rural/township vs. mixed); and quality score (≤5 vs. >5). Differences within each subgroup were compared using Cochran’s chi-squared test (Cochran’s Q).

To further explore the relevant factors influencing prevalence estimates, univariate meta-regression analysis was conducted including the following covariates: year of publication, income classification, sample size, response rate, method of data collection, professional group, region of health care setting, type of health care setting, and quality score. The multivariate meta-regression analysis included only significant variables (*p* < 0.05) in the regression model based on the result of the univariate analysis.

Publication bias was assessed by the Begg’s rank test, and a Begg’s funnel plot for a symmetry was presented. Sensitivity analysis was conducted by removing each study sequentially to assess the consistency of the prevalence estimates. All statistical analyses were two-tailed with a significance level of 0.05.

## 3. Results

### 3.1. Study Selection

**Figure 1:**
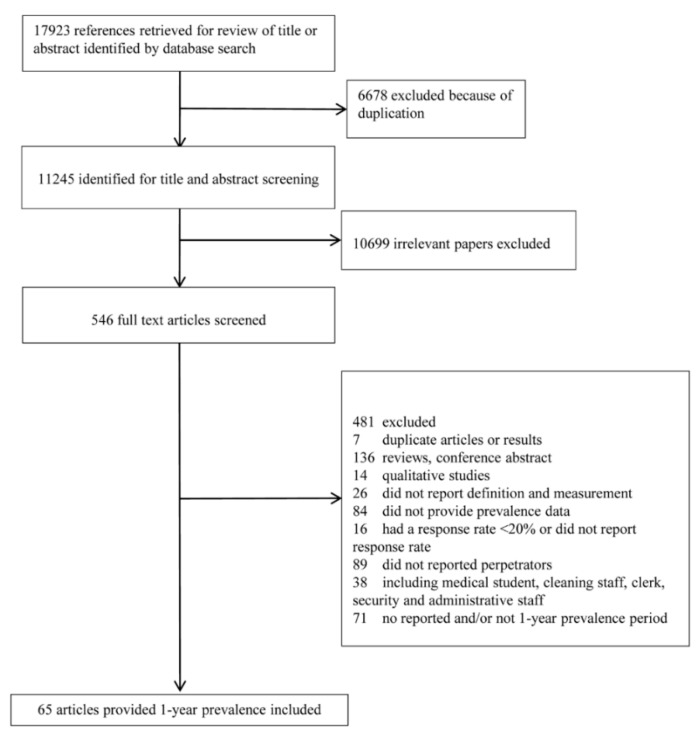
Preferred Reporting Items for Systematic Reviews and Meta-Analyses (PRISMA) flow chart of study identification process.

The database search initially generated 17,923 articles and 6678 duplicates were removed. After title and abstract screening, 10,699 irrelevant articles were excluded. A total of 546 potentially relevant full-text articles were independently assessed based on the selection criteria. Further, 481 studies were excluded because of the following reasons: duplicate articles or results (*n* = 7); reviews and conference abstracts (*n* = 136); used qualitative method only (*n* = 14); did not report definition or measurement (*n* = 26); had a response rate <20% or did not report response rate (*n* = 16); did not provide workplace physical violence prevalence data (*n* = 84); did not reported perpetrators (*n* = 89); including medical student, cleaning staff, clerk, security, and administrative staff (*n* = 38); and no reported and/or not one-year prevalence period (*n* = 71). Finally, 65 eligible studies were included for the meta-analysis (Figure 1).

### 3.2. Study Characteristics

A total of 61,800 health care professionals were included in this meta-analysis and the sample size ranged from 55 to 9218 participants per study. The eligible studies from 30 countries were geographically diverse, with 18 studies from the WHO region of Europe, 17 from the eastern Mediterranean, 14 from the western Pacific, 10 from the Americas, 4 from Africa, and 2 from Southeast Asia. Those countries were also divided into different income classification as follows: 32 studies from high-income countries, 20 from upper-middle-income countries, 11 from lower-middle-income countries, and 2 from low-income countries. Among eligible studies, 34 studies exclusively focused on nurses, 10 exclusively focused on physicians, and 16 focused on mixed staff categories. Quality scores ranged from three to eight points across studies. Minimum quality score of three was achieved in two studies and maximum quality score of eight was achieved in six studies. Seventeen studies were scored four points, 12 were scored five points, 18 were scored six points, and 10 were scored seven points (see Table 1).

### 3.3. Pooled One-Year Prevalence of Workplace Physical Violence

**Figure 2:**
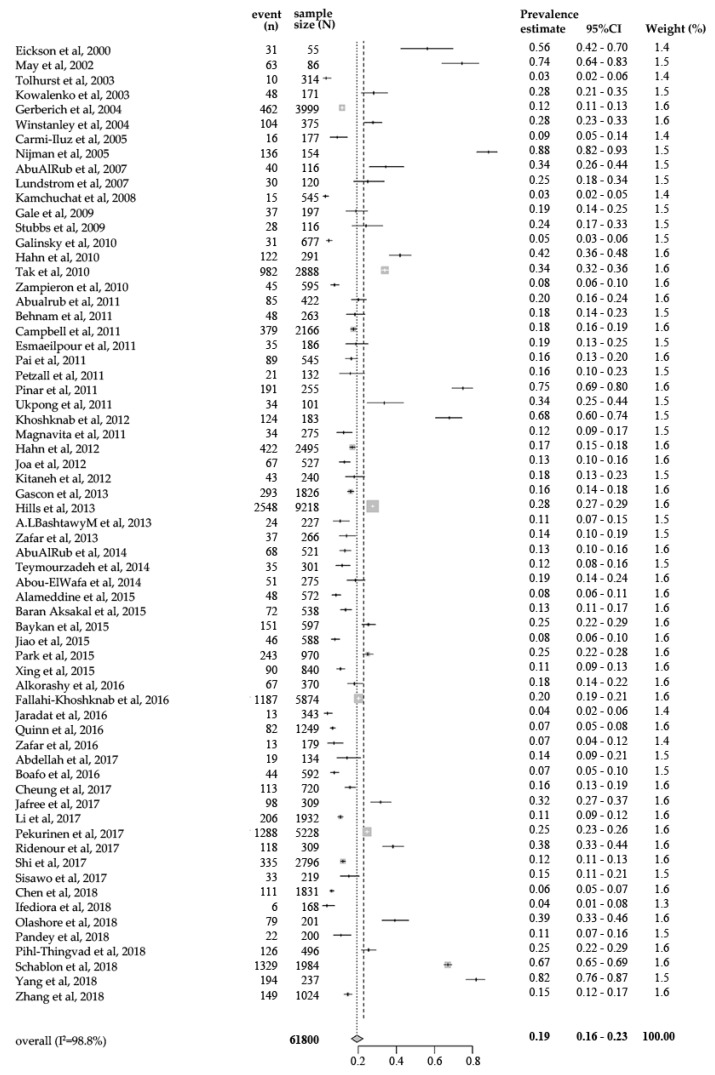
Forest plot of eligible studies. CI, confidence interval.

A total of 65 studies reported one-year prevalence of workplace physical violence against health care professionals perpetrated by patients or visitors, with prevalence estimates ranging from 2.75% to 88.31%. The lowest one-year prevalence was found among nurses in Thailand [16] and the highest was found among psychiatric nurses in the United Kingdom [30]. The pooled one-year prevalence of workplace physical violence against health care professionals perpetrated by patients or visitors was 19.33% (95% confidence interval (CI): 16.49–22.53%, Figure 2) by a random effects model. The analysis revealed significant heterogeneity between studies (I^2^ = 98.8%, *p* < 0.001).

### 3.4. Subgroup Analyses

For the regional level, pooled one-year prevalence of workplace physical violence against health care professionals perpetrated by patients or visitors was 26.38% (95% CI: 18.42–36.25%) in the European region, 23.61% (95% CI: 15.25–34.67%) in the Americas region, 20.71% (95% CI: 8.59–42.07%) in the African region, 17.07% (95% CI: 13.15–21.86%) in the eastern Mediterranean region, 14.53% (95% CI: 10.05–20.54%) in the Western Pacific region, and 5.62% (95% CI: 1.38–20.14%) in the Southeast Asia region. The pooled one-year prevalence of workplace physical violence against health care professionals perpetrated by patients or visitors in high-income, upper-middle-income, lower-middle-income, and low-income countries was 21.66% (95% CI: 17.49–26.51%), 19.98% (95% CI: 14.61–26.69%), 13.75% (95% CI: 9.49–19.50%), and 13.14% (95% CI: 9.62–%17.70%), respectively.

Prevalence estimates varied by health care facilities and staff categories. The pooled one-year prevalence estimates in tertiary hospital, secondary hospital, primary care facilities, and nursing home were 22.48% (95% CI: 15.35–31.69%), 18.83% (95% CI: 9.94–32.77%), 6.51% (95% CI: 4.36–9.64%), and 30.33% (95% CI: 22.32–39.75%), respectively. The pooled one-year prevalence of workplace physical violence against nurses perpetrated by patients or visitors was significantly higher than that against physicians (22.99% vs. 14.66%, *Q* = 4.38, *p* = 0.0364). Studies conducted in rural and township areas had significantly lower prevalence estimates than urban areas (6.11% vs. 26.16%, *Q* = 7.93, *p* = 0.0190). The pooled one-year prevalence of workplace physical violence against male health care professionals perpetrated by patients or visitors was similar to that against female health care professionals (7.37% vs. 8.40%, *Q* = 0.04, *p* = 0.8392).

Some methodological characteristics also influenced prevalence estimates across studies. When compared studies with sample size >500, studies with sample sizes ≤500 had higher prevalence estimates (13.96% vs. 24.48%, *Q* = 9.91, *p* = 0.0016). When compared studies with response rate >50%, studies with response rate ≤50% had higher prevalence estimates (17.65% vs. 38.53%, *Q* = 4.31, *p* = 0.0379). Subgroup analysis showed the sampling method, year of publication, and method for data collection were not statistically associated with prevalence estimates. All details about the subgroup analysis are provided in Table 2.

### 3.5. Meta-Regression Analyses

Bivariate meta-regression suggested higher prevalence estimates reported in studies with a smaller sample size (*β* = −0.698, *p* = 0.0098), in tertiary hospital (*β* = 1.470, *p* = 0.0022), and lower quality score (*β* = −0.213, *p* = 0.0364). Specifically, sample size accounted for 8.72% of the heterogeneity, type of the health care setting accounted for 14.20% of the heterogeneity, and quality score accounted for 5·41% of the heterogeneity across studies. Finally, sample size, type of health care setting, and quality score were entered into multivariate meta-regression model. Of the multivariate model, type of health care setting (*β* = 1.835, *p* = 0.0003) and quality score (*β* = −0.301, *p* = 0.0105) remained significant and accounted for 24.87% of the heterogeneity (Table 3).

### 3.6. Sensitivity Analysis and Publication Bias

**Figure 3:**
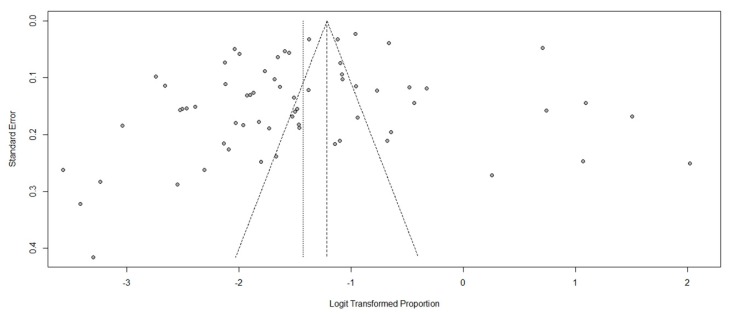
Funnel plots estimating small sample bias.

After one-by-one removals of 65 studies, the pooled one-year prevalence of workplace physical violence against health care professionals perpetrated by patients or visitors varied from 18.55% (95% CI: 15.82–21.63%) to 19.77% (95% CI: 16.87–23.03%), and the I^2^ statistic varied from 98.2% to 98.8%. The results of the sensitivity analysis revealed that no individual study significantly influenced the results. Publication bias was not observed in this meta-analysis, with the *p*-value for the Begg’s rank test being 0.1012 (Figure 3).

## 4. Discussion

Using meta-analytical methods, we pooled the one-year prevalence estimates of workplace physical violence against health care professionals perpetrated by patients or visitors reported in 65 studies published between 2000 and 2018. Eligible studies included 61,800 health care professionals from 30 countries. The one-year prevalence of workplace physical violence against health care professionals perpetrated by patients or visitors was 19.33% (95% CI: 16.49–22.53%) worldwide, or about one in five health care professionals annually. To the best of our knowledge, this study provided the first quantitative estimate of the prevalence of workplace physical violence against health care professionals perpetrated by patients or visitors worldwide.

Few review articles specifically focused on the prevalence of workplace physical violence against health care professionals perpetrated by patients or visitors. A systematic review conducted in 2008 found that, on average, 25% of health care professionals have experienced workplace physical violence perpetrated by patients or visitors in general hospital [82]. Another systematic review conducted in 2013 found that 2% to 32% hospital workers have experienced workplace physical violence perpetrated by patients or visitors [18]. Previous systematic review did not synthesize results by meta-analysis. This current meta-analysis revealed that 19.33% of health care professionals have experienced workplace violence perpetrated by patients or visitors worldwide annually. Our estimate of 19.33% was pooled based on 65 studies across all health care sectors, diverse health care professional types, and multiple countries. Prevalence estimates varied by region, with 26.38% in the European region, 23.61% in the Americas region, 20.71% in the African region, 17.07% in the eastern Mediterranean region, 14.53% in the western Pacific region, and 5.62% in the Southeast Asia region. Though eligible studies covered all WHO regions, prevalence studies were sparse in the Southeast Asia and African region. Besides, half of the eligible studies in this meta-analysis were conducted in high-income countries. More studies in low-income and lower-middle-income countries were needed.

Among eligible studies, more than half of the studies were published after 2010. We found that the year of publication was not associated with the prevalence estimates. Administrative strategies, preventive interventions, and policy against workplace violence have been advocated in health sectors over the decade [83,84]. A survey conducted by the National Crime Victimization suggested that the rate of nonfatal workplace violence has declined by 35% in the United States from 2002 to 2009 [3]. In the present study, the one-year prevalence estimate was not significantly declined worldwide based on the result of the subgroup analysis. Practical intervention in health care sectors was still an urgent need. Our finding may vary with geographical location because each country had its own special working environment and conditions. Future research could benefit from examining the national time trend of workplace physical violence and exploring how country-specific social factors and policy affected it.

The results revealed that studies with sample sizes ≤ 500 and studies with low response rate had significantly higher one-year prevalence estimates. The studies’ characteristics obviously influenced prevalence estimates of workplace physical. Studies with fewer participants generally yielded more extreme prevalence estimates [85], which may be attributed to selection bias and publication bias. Studies with a low response rate provided higher prevalence estimates as a result of report bias. In a meta-analysis of elder abuse, the result also suggested studies with small sample sizes were more likely to produce higher prevalence estimates [86].

The result of the subgroup analysis suggested that nurses experienced more workplace physical violence perpetrated by patients or visitors than physicians. This phenomenon was supported by numerous epidemiological studies [28,69,87,88]. Another meta-analysis also emphasized the disparate workplace physical violence experiences in nurses and physicians [22]. The working content and duties were quite different between nurses and physicians, as well as nurse–patient interaction and physician–patient interaction [69]. Nurses experiencing more physical violence may account for their gender, occupational prestige, and closer contact with patients and visitors [88]. Besides, as physicians dominated the process of treatment, patients or visitors might show more obedience and respect to physicians. We noticed that most of studies were specifically assessed workplace physical violence against nurses, while evidence of physicians was relatively limited. If more evidence of workplace physical violence against physicians was available, the finding of professional imparity might be more credible.

Gender difference was not observed in this meta-analysis. In a systematic review emphasizing gender difference of physical violence, the researcher found that numerous studies showed male health care professionals experienced more workplace physical violence than females [89]. This systematic review also suggested that 19 studies revealed a non-significant association between workplace physical violence and gender [89]. In this meta-analysis, only three eligible studies reported rates of workplace physical violence perpetrated by patients or visitors for male and female health care professionals separately, which yielded a very limited result. Gender difference of workplace physical violence against health care professionals was an undetermined issue. It is necessary for future research to provide gender-specific prevalence estimates. Those studies could help us understand demographic characteristic of victims and provide evidences for well-targeted intervention.

Subgroup analyses revealed that health care professionals working in nursing homes experienced more physical violence from patients or visitors than those in other health care settings. Patients with dementia or disability in nursing homes might present more aggressive behavior and physical violence against health care professionals than general patients [37]. Except nursing homes, health care professionals working in tertiary hospitals experienced more workplace physical violence than those working in primary care facilities. To date, only a handful studies compared workplace physical violence between different health care settings. Gascon et al. found that health workers in a large hospital experience more physical violence than those in a small hospital and primary health center [52]. The risk factors of workplace physical violence such as overcrowding, noisy, long waiting time, and short consultation time occurred more frequently in tertiary hospitals [13,53,74,90]. Additionally, medical conditions of patients were generally severer in tertiary hospitals than those in primary care facilities. However, patients had higher expectations in tertiary hospitals than in primary care facilities [90,91]. Thus, patients might show less satisfaction and more aggression in tertiary hospitals. Clearly, the scant evidence suggested a need for further research exploring the role of health care settings.

Health care professionals working in rural or township areas experienced less workplace physical violence perpetrated by patients or visitors than those in urban areas. Few studies had emphasized the disparity between urban and rural areas [74]. Patients with a severe condition and high demand were prone to seek help directly in a tertiary hospital located in urban areas [92]. Thus, health care professionals were faced with more stressful working environment in urban areas, which increased the risk of workplace violence [13]. Research of violence also suggested that urban–rural disparity may be explained by social factors such as inequality and poor social cohesion [11]. Here, only two eligible studies specifically evaluated the prevalence of workplace physical violence against health care professionals perpetrated by patients or visitors in a rural or township area. More studies were needed to obtain a reliable estimate.

There were several limitations in the present study. First, although our study included relevant studies across 30 countries, half of the eligible studies were from high-income countries. Prevalence studies were scarce for many countries, especially for lower-middle-income and low-income countries. Considering the inconsistency of the health care environment and working conditions across the world, more prevalence studies in low-income and lower-middle-income countries are needed to understand the panorama of workplace physical violence against health care professionals. Second, the ability to compare findings and understand the magnitude of pooled prevalence was severely hampered by inconsistent methodology between studies, including inconsistent definitions, response rate, and methods of data collection. Although we have excluded those studies without description of definition and measurement, inconsistency was still inescapable. Numerous studies adopted self-designed and self-administrated questionnaire to measure workplace physical violence. It is hard to compare findings without a standard assessment tool. Thus, future research should develop a standard and comprehensive used assessment tool to measure workplace physical violence.

## 5. Conclusions

The pooled one-year prevalence of workplace physical violence against health care professionals perpetrated by patients or visitors was 19.33% (95% CI: 16.49–22.53%). About one in five health care professionals experienced workplace physical violence perpetrated by patients or visitors annually. One-year prevalence estimates varied significantly regarding the country of study, sample size, response rate, professional group, region of health care setting, and type of health care setting. Significant moderators for heterogeneity included sample size, type of health care setting, and quality score. Future research can benefit from exploring gender differences, occupational differences, and time trends in workplace physical violence against health care professionals. More practical intervention and policy defensed workplace physical violence were needed to ensure the safety of health care professionals.

## Figures and Tables

**Table 1:** Characteristics of prevalence studies included in meta-analysis. WHO, World Health Organization.

Study	Country	WHO Region	Sample Size	Event	Income Classification	Professional Group	Setting	Region of Health Care Setting	Method of Data Collection	Response Rate	Sampling	Quality Score
Eickson et al., 2000 [24]	U.S.	Americas	55	31	High income	nurses	emergency department	urban	self-administered	98.00%	convenience	4
May et al., 2002 [17]	U.S.	Americas	86	64	High income	nurses	general hospital	urban	self-administered	68.80%	convenience	3
Tolhurst et al., 2003 [25]	Australia	Western Pacific	314	10	High income	physicians	primary care	rural	self-administered	51.80%	purposive	4
Kowalenko et al., 2003 [26]	U.S.	Americas	171	48	High income	physicians	emergency department	mixed	self-administered	68.40%	random	4
Gerberich et al., 2004 [27]	U.S.	Americas	3999	462	High income	nurses	Hospital/nursing home/other setting	mixed	self-administered	78.00%	random	6
Winstanley et al., 2004 [28]	U.K.	European	375	104	High income	nurses/physicians	general hospital	mixed	self-administered	33.00%	all	6
Carmi-Iluz et al., 2005 [29]	Israel	European	177	16	High income	physicians	hospital/community	mixed	self-administered	88.50%	convenience	4
Nijman et al., 2005 [30]	U.K.	European	154	136	High income	nurses	psychiatric	urban	self-administered	39.00%	all	4
AbuAlRub et al., 2007 [31]	Iraq	Eastern Mediterranean	116	40	Upper middle income	nurses	general hospital	urban	face-to-face interview	100.00%	purposive	5
Lundstrom et al., 2007 [32]	Sweden	European	120	30	High income	nurses/assistant nurses/nurse’s aides	nursing home	urban	self-administered	81.00%	/	4
Kamchuchat et al., 2008 [16]	Thailand	South-East Asia	545	15	Upper middle income	nurses	general hospital	/	self-administered	91.70%	all	7
Gale et al., 2009 [33]	New Zealand	Western Pacific	197	37	High income	physicians	psychiatric	mixed	self-administered	63.90%	all	4
Stubbs et al., 2009 [34]	U.K.	European	116	28	High income	physicians	psychiatric	mixed	self-administered	65.00%	all	4
Galinsky et al., 2010 [35]	U.S.	Americas	677	31	High income	nurses/assistant nurses/nurse’s aides	home healthcare	urban	face-to-face interview	64.00%	convenience	4
Hahn et al., 2010 [36]	Switzerland	European	291	122	High income	nurses	general hospital	/	self-administered	71.00%	purposive	4
Tak et al., 2010 [37]	U.S.	Americas	2888	982	High income	assistant nurses	nursing home	/	telephone interview	70.60%	random	7
Zampieron et al., 2010 [38]	Italy	European	595	45	High income	nurses	general hospital	urban	self-administered	85.00%	convenience	6
Abualrub et al., 2011 [39]	Jordan	Eastern Mediterranean	422	85	Upper middle income	nurses	general hospital	/	self-administered	84.40%	convenience	5
Behnam et al., 2011 [40]	U.S.	Americas	263	48	High income	physicians	emergency department	mixed	self-administered	97.00%	random	6
Campbell et al., 2011 [41]	U.S.	Americas	2166	379	High income	nurses	hospital/elder care	urban	self-administered	52.00%	all	5
Esmaeilpour et al., 2011 [42]	Iran	Eastern Mediterranean	186	35	Upper middle income	nurses	emergency department	urban	self-administered	94.80%	/	6
Pai et al., 2011 [43]	China Taiwan	Western Pacific	545	89	High income	nurses	health care setting	mixed	self-administered	77.90%	random	5
Petzall et al., 2011 [44]	Switzerland	European	132	21	High income	ambulance personnel	ambulance stations	mixed	self-administered	79.00%	convenience	8
Pinar et al., 2011 [45]	Turkey	European	255	191	Upper middle income	nurses	emergency department	urban	self-administered	96.22%	all	4
Ukpong et al., 2011 [46]	Nigeria	African	101	34	Lower middle income	nurses/physicians	psychiatric	urban	self-administered	84.20%	/	5
Khoshknab et al., 2012 [47]	Iran	Eastern Mediterranean	183	124	Upper middle income	nurses	psychiatric	urban	self-administered	91.50%	random	4
Magnavita et al., 2011 [48]	Italy	European	275	34	High income	nurses	general hospital	/	self-administered	94.20%	/	6
Hahn et al., 2012 [49]	Switzerland	European	2495	422	High income	health care professionals	general hospital	/	self-administered	51.50%	all	4
Joa et al., 2012 [50]	Norway	European	527	67	High income	health care professionals	primary care	mixed	telephone interview	75.00%	all	6
Kitaneh et al., 2012 [51]	Palestine	Eastern Mediterranean	240	43	Lower middle income	nurses/physicians	general hospital	/	self-administered	88.70%	random	6
Gascon et al., 2013 [52]	Spain	European	1826	293	High income	health care professionals	hospital/primary care center	mixed	self-administered	76.00%	random	6
Hills et al., 2013 [53]	Australia	Western Pacific	9218	2548	High income	physicians	health care setting	mixed	self-administered	60.90%	all	7
A.LBashtawyM et al., 2013 [54]	Jordan	Eastern Mediterranean	227	24	Lower middle income	nurses	emergency department	/	self-administered	72.50%	convenience	6
Zafar et al., 2013 [55]	Pakistan	Eastern Mediterranean	266	37	Lower middle income	nurses/physicians	emergency department	urban	self-administered	86.00%	all	4
AbuAlRub et al., 2014 [56]	Jordan	Eastern Mediterranean	521	68	Lower middle income	nurses/physicians	general hospital	/	self-administered	75.00%	all	6
Teymourzadeh et al., 2014 [57]	Iran	Eastern Mediterranean	301	35	Upper middle income	nurses	general hospital	urban	self-administered	73.00%	all	8
Abou-ElWafa et al., 2014 [58]	Egypt	Eastern Mediterranean	275	51	Lower middle income	nurses	Emergency department/internal medicine	urban	self-administered	96.15%	all	8
Alameddine et al., 2015 [8]	Lebanon	Eastern Mediterranean	572	48	Upper middle income	nurses	health care setting	/	self-administered	64.80%	random	6
Baran Aksakal et al., 2015 [59]	Turkey	European	538	72	Upper middle income	nurses	general hospital	/	face-to-face interview	82.76%	all	6
Baykan et al., 2015 [60]	Turkey	European	597	151	Upper middle income	physicians	health care workplace	/	self-administered	75.90%	all	8
Jiao et al., 2015 [61]	China	Western Pacific	588	46	Upper middle income	nurses	general hospital	urban	self-administered	84.00%	random	7
Park et al., 2015 [62]	Korea	Western Pacific	970	243	High income	nurses	general hospital	urban	self-administered	95.20%	convenience	8
Xing et al., 2015 [63]	China	Western Pacific	840	90	Upper middle income	nurses/physicians	primary care	rural	self-administered	84.80%	purposive	5
Alkorashy et al., 2016 [64]	Saudi Arabia	Eastern Mediterranean	370	67	High income	nurses	general hospital	urban	self-administered	80.80%	convenience	6
Fallahi-Khoshknab et al., 2016 [65]	Iran	Eastern Mediterranean	5874	1187	Upper middle income	health care professionals	general hospital	/	self-administered	90.36%	random	5
Jaradat et al., 2016 [9]	Palestine	Eastern Mediterranean	343	13	Lower middle income	nurses	hospitals/primary care	/	self-administered	92.20%	/	8
Quinn et al., 2016 [66]	U.S.	Americas	1249	82	High income	home care aides	home healthcare	/	self-administered	44.20%	/	6
Zafar et al., 2016 [7]	Pakistan	Eastern Mediterranean	179	13	Lower middle income	physicians	general hospital	urban	self-administered	92.20%	all	4
Abdellah et al., 2017 [67]	Egypt	Eastern Mediterranean	134	19	Lower middle income	Health care professionals	emergency department	urban	self-administered	94.40%	/	7
Boafo et al., 2016 [68]	Ghana	African	592	44	Lower middle income	nurses	general hospital	/	self-administered	57.98%	random	4
Cheung et al., 2017 [69]	China	Western Pacific	720	113	Upper middle income	nurses/physicians	general hospital	urban	self-administered	80.00%	convenience	7
Jafree et al., 2017 [70]	Pakistan	Eastern Mediterranean	309	98	Lower middle income	nurses	general hospital	urban	self-administered	34.80%	random	6
Li et al., 2017 [71]	China	Western Pacific	1932	206	Upper middle income	nurses/physicians	pediatric hospital	urban	self-administered	86.80%	random	7
Pekurinen et al., 2017 [72]	Finland	European	5228	1288	High income	nurses	general hospital	/	self-administered	70.00%	all	7
Ridenour et al., 2017 [73]	U.S.	Americas	309	118	High income	nurses	general hospital	/	self-administered	22.50%	random	7
Shi et al., 2017 [6]	China	Western Pacific	2796	335	Upper middle income	health care professionals	general hospital	/	self-administered	64.25%	convenience	6
Sisawo et al., 2017 [74]	Gambia	African	219	33	low income	nurses	general hospital	mixed	face-to-face interview	98.20%	purposive	5
Chen et al., 2018 [75]	China	Western Pacific	1831	111	Upper middle income	nurses	general hospital	urban	self-administered	92.30%	all	5
Ifediora et al., 2018 [76]	Australia	Western Pacific	168	6	High income	physicians	primary care	/	self-administered	56.00%	/	7
Olashore et al., 2018 [77]	Botswana	African	201	79	Upper middle income	health care professionals	psychiatric	urban	self-administered	95.70%	all	3
Pandey et al., 2018 [78]	Nepal	South-East Asia	200	22	low income	nurses	general hospital	/	self-administered	100.00%	random	5
Pihl-Thingvad et al., 2018 [15]	Denmark	European	496	126	High income	health care professionals	general hospital	urban	self-administered	28.00%	all	7
Schablon et al., 2018 [79]	Germany	European	1984	1329	High income	nurses	hospital/elder care/residential facility	/	self-administered	40.90%	random	6
Yang et al., 2018 [80]	China	Western Pacific	237	194	Upper middle income	nurses	psychiatric	urban	self-administered	84.50%	/	5
Zhang et al., 2018 [81]	China	Western Pacific	1024	149	Upper middle income	nurses	general hospital	/	self-administered	75.18%	snowball	4

**Table 2:** Subgroup analysis of the pooled prevalence.

Subgroup	Studies	Pooled Prevalence % (95% CI)	I^2^	Test of Difference within Each Subgroup	
				*Q*	*p*
WHO Region				10.60	0.0599
European	18	26.38 (18.42–36.25)	99.2%		
Americas	10	23.61 (15.25–34.67)	99.0%		
African	4	20.71 (8.59–42.07)	97.3%		
Eastern Mediterranean	17	17.07 (13.15–21.86)	95.7%		
Western Pacific	14	14.53 (10.05–20.54)	98.9%		
South-East Asia	2	5.62 (1.38–20.14)	94.5%		
Income classification				9.84	0.0020
High-income	32	21.66 (17.49–26.51)	99.0%		
Upper-middle-income	20	19.98 (14.61–26.69)	98.7%		
Lower-middle-income	11	13.75 (9.49–19.50)	93.6%		
Low-income	2	13.14 (9.62–17.70)	33.6%		
Year of publication				1.06	0.3036
2000–2010	17	22.83 (15.31–32.61)	98.6%		
2011–2018	48	18.22 (15.17–21.73)	98.8%		
Sample size				9.91	0.0016
≤500	37	24.48 (18.84–31.16)	97.2%		
>500	28	13.96 (10.99–17·57)	99.3%		
Response rate				4.31	0.0379
≤50%	7	38.53 (18.75–63.00)	99.4%		
>50%	58	17.65 (15.33–20.23)	98.1%		
Professional group				4.38	0.0364
nurses	34	22.99 (17.11–30.16)	99.1%		
physicians	10	14.66 (10.67–19.81)	94.4%		
Method of collection				0.88	0.6441
Self-administered	59	19.66 (16.60–23.14)	98·8%		
face-to-face interview	4	13.93 (6.39–27.76)	96.3%		
Telephone interview	2	21.61 (7.40–48.73)	98.8%		
Gender				0.04	0.8392
Male	3	7.37 (2.00–23.69)	89.5%		
Female	3	8.40 (6.72–10.46)	37.9%		
Sampling				0.84	0.6572
all	21	20.82 (16.89–25.38)	98.2%		
Random	17	20.86 (14.19–29.59)	99.4%		
convenience	17	17.64 (12.82–23.79)	97.1%		
Region of health care setting				7.93	**0.0190**
Urban	27	26.16 (19.11–34.69)	98.5%		
Rural/township	2	6.11 (1.80–18.76)	93.1%		
Mixed	13	17.85 (13.68–22.97)	97.7%		
Type of health care setting				39.52	**<0.0001**
Tertiary hospital	18	22.48 (15.35–31.69)	98.4%		
Secondary hospital	3	18·83 (9.94–32.77)	91.3%		
Primary care facilities	6	6.51 (4.36–9.64)	90.2%		
nursing home	2	30.33 (22.32–39.75)	75.7%		
Quality score				3.92	**0.0476**
≤5	31	24.00 (16.41–33.69)	99.0%		
>5	34	15.86 (13.53–18.51)	98.3%		

Bold values are significant (*p* < 0.05).

**Table 3:** Meta-regression analyses of the effects of potential moderators.

Univariate Analysis	*β*	95% CI		R^2^	*p* Value
		Lower	Upper		
Publish year (continuous variable)	−0.0483	−0.1076	0.0109	2.29%	0.1100
Sample size (*n* < 500 vs. *n* ≥ 500)	−0.6983	−1.2281	−0.1685	8.72%	**0.0098**
Response rate (continuous variable)	−0.7139	−2.1540	0.7262	0.00%	0.3313
Income Classification (high income vs. other)	0.2798	−0.2698	0.8294	0.00%	0.3183
Professional (nurses vs. other)	−0.6344	−1.5518	0.2831	1.66%	0.1753
Region of health care setting (urban vs. rural)	0.6527	−0.0754	1.3808	4.78%	0.0789
Type of health care setting (tertiary hospital vs. primary care facilities)	1.4696	0.5297	2.4095	14.20%	**0.0022**
Method of data collection (Self-administered vs. others)	0.4451	−0.1229	1.0130	2.22%	0.1245
Quality score (continuous variable)	−0.2125	−0.4117	−0.0134	5.41%	**0.0364**
Multivariate Analysis					
Sample size (*n* < 500 vs. *n* ≥ 500)	−0.1671	−0.7712	0.4369	/	0.5876
Type of health care setting (tertiary hospital vs. primary care facilities)	1.8345	0.8373	2.8316	/	**0.0003**
Quality score (continuous variable)	−0.3008	−0.5314	−0.0703	/	**0.0105**
Overall				24.87%	

Bold values are significant (*p* < 0.05).

## Supplementary Materials

The following are available online at https://www.mdpi.com/1660-4601/17/1/299/s1, Table S1: PRISMA-2009-checklist; Table S2: Database searches results; Table S3: Methodological quality assessment of the eligible studies.
